# Probing the shRNA characteristics that hinder Dicer recognition and consequently allow Ago-mediated processing and AgoshRNA activity

**DOI:** 10.1261/rna.043950.113

**Published:** 2014-09

**Authors:** Elena Herrera-Carrillo, Alex Harwig, Ying Poi Liu, Ben Berkhout

**Affiliations:** 1 Laboratory of Experimental Virology, Department of Medical Microbiology, Center for Infection and Immunity Amsterdam (CINIMA), Academic Medical Center, University of Amsterdam, 1105 AZ, Amsterdam, The Netherlands

**Keywords:** RNAi, Dicer, Ago2, shRNA design, Dicer-independent shRNA, miR-451

## Abstract

Recent evidence indicates the presence of alternative pathways for microRNA (miRNA) and short hairpin (shRNA) processing. Specifically, some of these molecules are refractory to Dicer-mediated processing, which allows alternative processing routes via the Ago2 endonuclease. The resulting RNA molecules differ in size and sequence and will thus trigger the silencing of different target RNAs. It is, therefore, important to understand these processing routes in mechanistic detail such that one can design exclusive RNA reagents for a specific processing route. The exact sh/miRNA properties that determine this routing toward Dicer or Ago2 are incompletely understood. The size of the base-paired stem seems an important determinant, but other RNA elements may contribute as well. In this study, we document the importance of a weak G-U or U-G base pair at the top of the hairpin stem.

## Abstract (precis)

Although most microRNAs (miRNAs) and short hairpin RNAs (shRNAs) are processed by Dicer, unusual Dicer-independent miRNAs and shRNAs have recently been described that are processed by Ago2. A relatively short stem (<19 bp) in the substrate helps to specify this alternative processing route, but additional determinants remain to be identified. Here, the authors demonstrate that a specific G-U or U-G base-pairing arrangement at the top of the hairpin stem is important for Ago2-mediated shRNA processing.

## INTRODUCTION

Small noncoding microRNAs (miRNAs) regulate cellular gene expression at the post-transcriptional level (Brummelkamp et al. 2002; Bartel 2009; Carthew and Sontheimer 2009). These miRNAs are usually processed by the nuclear Drosha endonuclease, transported to the cytoplasm by Exportin-5, further processed by the Dicer endonuclease, and subsequently associate with an Argonaute (Ago) protein in the RNA-induced silencing complex (RISC) to induce degradation of complementary target RNAs (Filipowicz et al. 2008; Siomi and Siomi 2009). This RNA interference (RNAi) mechanism can also be triggered by man-made gene constructs that express short hairpin RNA (shRNA) molecules in the nucleus that enter the RNAi pathway at the Dicer processing step in the cytoplasm, followed by silencing of the complementary mRNA target. However, there is a growing body of evidence for the existence of noncanonical or alternative processing routes for natural miRNAs and man-made shRNAs. Whereas Dicer is critical for activation of the majority of these miRNA and shRNA molecules, notable exceptions were reported for a special set of miRNAs (Cheloufi et al. 2010; Cifuentes et al. 2010; Miyoshi et al. 2010; Yang et al. 2010; Yang and Lai 2011; Havens et al. 2012). More recently, Dicer-independent shRNAs have been described as well (Ge et al. 2010; Dallas et al. 2012; Liu et al. 2013). We called these molecules AgoshRNAs because Ago2 is involved in their processing (Liu et al. 2013).

**FIGURE 1.**
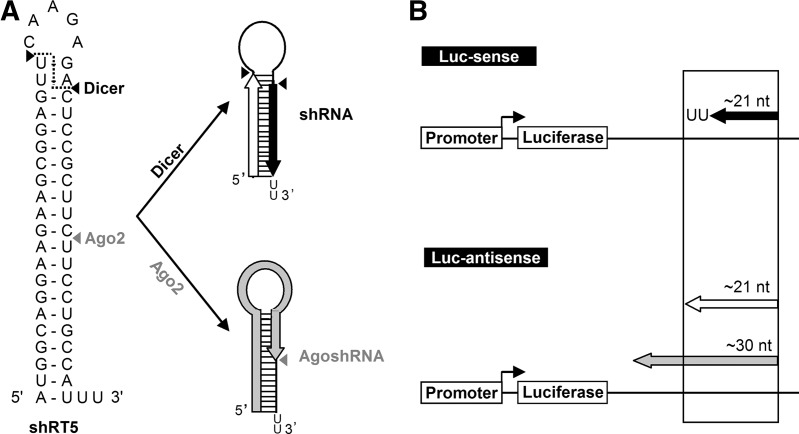
Schematic of canonical (Dicer-dependent) and noncanonical (Ago2-dependent) shRNA processing routes. (*A*) Secondary structure of a regular shRNA (shRT5) as predicted by Mfold. In the canonical pathway, the stem of the shRNA is cleaved by Dicer into a siRNA duplex of ∼21 bp with 3′ overhangs that is loaded into the RISC. One strand (the passenger, white arrow) is cleaved and degraded; the other acts as guide (black arrow) in RNAi-silencing. Alternatively, the shRNA can be recognized directly by Ago2 such that it is cleaved on the 3′ side between base pairs 10 and 11 into a single-stranded RNA molecule of ∼30 nt (gray arrow), which subsequently instructs Ago2 for RNAi-silencing. This is the AgoshRNA route. The predicted Dicer and Ago2 cleavage sites are marked with black and gray arrows, respectively. (*B*) Luc reporter constructs with sense and anti-sense HIV-derived sequences. The Luc-sense reporter scores canonical shRNA guide activity; the Luc-anti-sense reporter scores both shRNA passenger and AgoshRNA activity.

It seems important to understand these Dicer-independent processing routes, as quite different RNA species are generated by Ago-mediated processing, such that a totally different (set of) mRNA(s) will be targeted. Figure 1 depicts these two processing routes for a regular shRNA substrate, which lead to remarkably different short RNA species that trigger the RNAi response. Regular Dicer-cleavage of both strands of the base-paired stem near the loop region generates the small interfering RNA (siRNA), consisting of two candidate active strands of ∼21 nucleotides (nt), marked with a black arrow for the guide strand and white arrow for the passenger strand (Fig. 1A, top). The activity of these two strands can be scored by silencing of the Luc-sense or Luc-anti-sense reporter, respectively (Fig. 1B). As our shRNA constructs were initially developed as new anti-viral reagents against the human immunodeficiency virus (HIV) RNA genome (Ter Brake et al. 2006, 2008), the sense reporter encodes HIV sequences and the anti-sense reporter, the complementary strand. The alternative Dicer-independent processing route is depicted in Figure 1A, bottom. Ago2-mediated cleavage on the 3′ side of the hairpin between base pairs (bp) 10 and 11 generates an extended RNA molecule of ∼30 nt that can anneal exclusively to the Luc-anti-sense reporter (Fig. 1B).

A detailed mutational analysis indicated that the length of the base-paired stem is the major determinant for shRNA activity via the regular Dicer route vs. AgoshRNA activity via the noncanonical Ago2 route (Liu et al. 2013). shRNAs of 19 bp or less lose the ability to be processed by Dicer, which opens up the alternative Ago2-processing route for AgoshRNAs. AgoshRNAs remain active down to 17 bp, but shorter hairpins lose all activity (Liu et al. 2013). However, other sequence and/or structure elements may also influence the routing of shRNAs. In this study, we zoom in on the identity of the top base pair in AgoshRNAs as a candidate determinant for Dicer-independent processing.

Two findings hinted at the importance of the top base pair. First, Dueck et al. recently suggested that the top G-U base pair in miR-451 is important for activation of the Dicer-independent processing route (Dueck et al. 2012). Specifically, mutation to G-C reduced the Ago2-mediated processing efficiency, suggesting that miR-451 was evolutionary optimized for this alternative processing route. Second, one of the hallmarks of the AgoshRNA molecules is the presence of a top U-G base pair that was originally designed to be part of the single-stranded loop (Brummelkamp et al. 2002) but that, in fact, extends the hairpin stem with two additional base pairs (Schopman et al. 2010). The original shRNA design by Brummelkamp et al. has a single-stranded loop of 9 nt, but closer inspection revealed the possibility to form two additional base pairs, U-A and U-G, as the closing pair, yielding a loop of 5 nt (Fig. 1A). We wondered whether the presence of the weak U-G as the ultimate base pair influences the shift from regular shRNA activity to noncanonical AgoshRNA activity, possibly because Dicer interrogates the stability of the ends of the base-paired stem region. In this study, we set out to specifically test the contribution of the terminal base pair on shRNA vs. AgoshRNA activity.

## RESULTS

### A top G-U base pair in a shRNA series with different stem length

**FIGURE 2.**
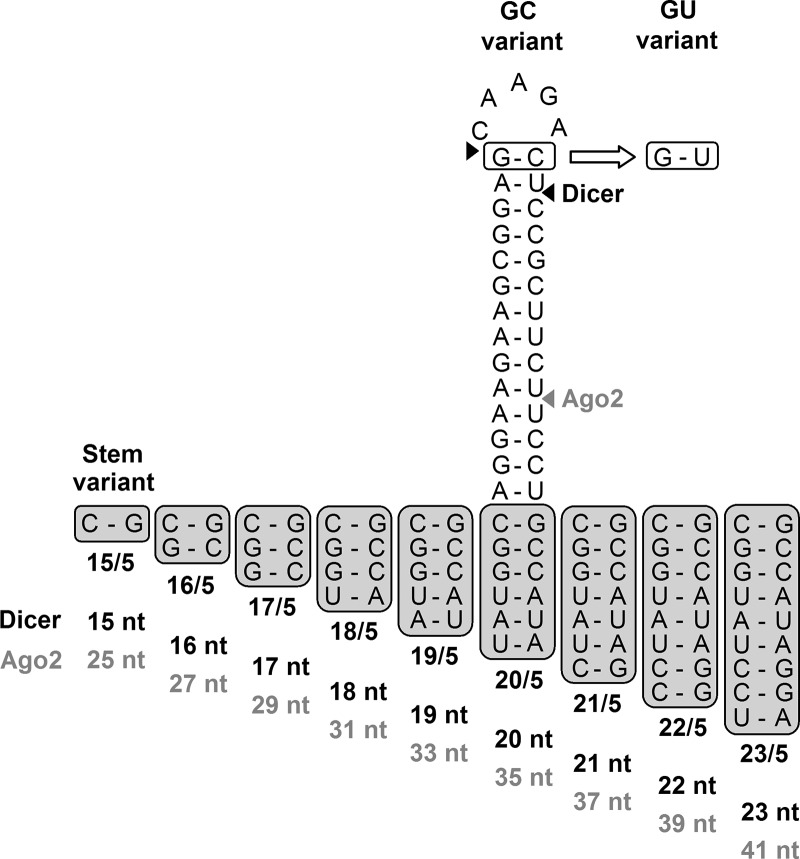
Design of shRT5 mutants with a terminal G-C or G-U base pair and varying stem length. The shRT5 with a 19-bp stem and 5-nt loop (19/5) was used as template. The shRNA stem length was reduced/increased at the bottom of the hairpin, resulting in shRT5 variants 15/5–23/5 that do not affect the complementarity with the probe used in Northern blotting. In this shRNA length series, the terminal top base pair G-C was modified to G-U. The predicted Dicer and Ago2 cleavage sites are marked with black and gray arrows, respectively. Predicted RNA fragment lengths upon Ago2 and Dicer processing are shown *below* the structures.

**FIGURE 3.**
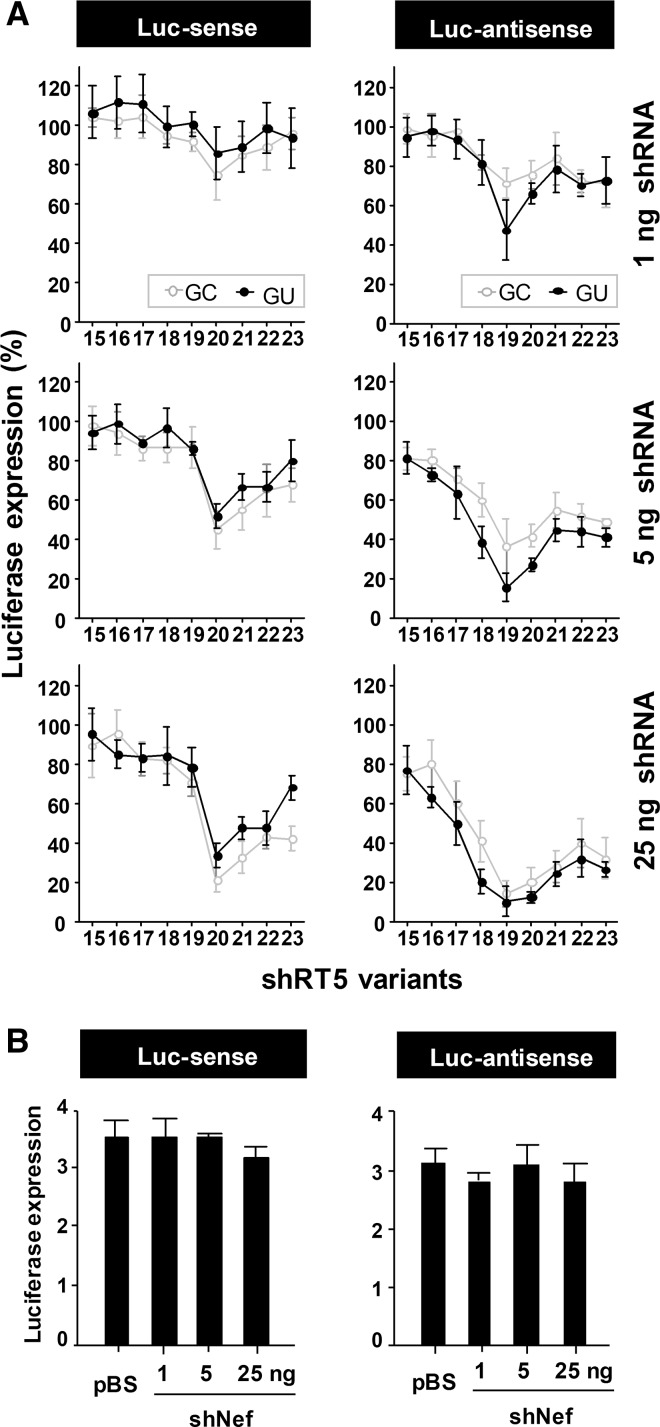
Knockdown activity of the shRT5 variants. (*A*) The knockdown activity of the different G-C and G-U shRNA variants was determined by cotransfection with a luciferase reporter containing either the sense- or anti-sense-target sequence. HEK 293T cells were cotransfected with 100 ng of the respective firefly luciferase reporter plasmid, 1 ng *Renilla* luciferase plasmid as an internal control, and 1, 5, or 25 ng of the corresponding shRNA construct. An irrelevant shRNA (shNef) served as a negative control; this activity was set at 100% luciferase expression. We performed three independent transfections, each in duplicate, and standard deviations were calculated. (*B*) Results obtained with the negative controls, the empty vector pBS, and the nonspecific shNef. See above for further details.

The first set of mutants was designed to probe the effect of a terminal G-C vs. G-U base pair in shRNAs ranging from 15 to 23 bp in length (Fig. 2). The additional base pairs were chosen based on extension of the complementarity with the HIV-1 RNA genome. For some constructs, this strategy could affect the transcription start site and the transcriptional efficiency from the H1 promoter (Li et al. 2007), although other studies reported no effect on the shRNA-mediated gene silencing efficiency (Tuschl 2002). The size range was chosen to encompass candidate AgoshRNAs (17–19 bp) and shRNAs (20 bp and above). We named the shRNAs according to the stem length and loop size, e.g., a shRNA with a stem length of 20 bp and a loop sequence of 5 nt is termed 20/5. Because of the different strand being activated during regular shRNA processing by Dicer vs. alternative processing by Ago2, we used two luciferase reporters with complementary target sequences to measure shRNA and AgoshRNA activity. The HIV-derived target sequences in these reporters were designed to be fully complementary to all shRNA/AgoshRNA variants used in this study. Please note that the Luc-anti-sense reporter detects AgoshRNA activity but also passenger strand activity of the regular Dicer-route (Fig. 1B). Let us first discuss the Dicer-route toward shRNA activity on the Luc-sense reporter and then the Ago2-route toward AgoshRNA activity on the Luc-anti-sense reporter. The reporter construct was cotransfected with 1, 5, or 25 ng of the shRNA constructs in HEK 293T cells. A fixed amount of *Renilla* luciferase plasmid was included as a control for the transfection efficiency. An irrelevant shRNA (shNef) served as a negative control, which showed no effect even up to 25 ng of construct. These values represent maximal, unhindered luciferase expression and were set at 100% (Fig. 3B). Little Luc-sense silencing is apparent for the short shRNAs, but a steep increase in shRNA activity and thus reduced luciferase expression is observed for hairpins of at least 20 bp (Fig. 3A, left panels). The even larger shRNAs do gradually lose activity. The G-C and G-U versions show approximately the same activity pattern, but the G-C variants seem the better inhibitors.

A narrow AgoshRNA activity window is observed on the Luc-anti-sense reporter for molecules in the size range of 17–20 bp (Fig. 3A, right panels). A more quantitative analysis is, however, difficult because a shift in the actual Dicer cleavage site (see Fig. 2) will minimally affect the complementarity with the Luc-sense reporter. Most importantly, we measured reduced luciferase expression and thus improved AgoshRNA activity for the G-U variants. Although the effects may seem small, they are quite striking when measured in the linear range of the assay with 5 ng of the shRNA construct. For instance, we measured 37% and 42% luciferase activity for the G-C hairpins of 19 and 20 bp, respectively, but only 16% and 27% luciferase expression was scored for the G-U versions. The larger hairpins (21–23 bp) maintained quite a lot of activity on the Luc-anti-sense reporter, but this is, in part, due to passenger strand activity of the regular shRNA-route. These combined results provide the first evidence that a weak top base pair may, indeed, favor AgoshRNA processing over the regular shRNA-route.

**FIGURE 4.**
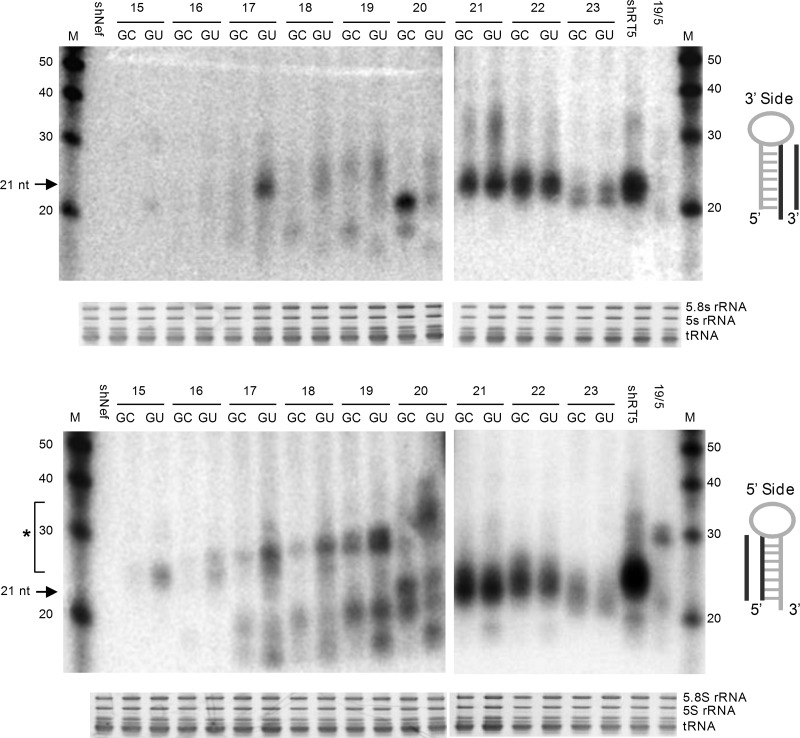
Processing of shRNAs is influenced by the stability of the top base pair and stem length. HEK 293T cells were transfected with 5 μg of the indicated shRNA constructs. The shRNAs varied in stem length and top base pair (G-C or G-U). Total RNA was isolated and analyzed by Northern blot using an LNA probe (see cartoon next to blot) to detect processing products derived from the 3′ strand (*upper* panel) and the 5′ strand (*lower* panel). Small differences in probe-transcript complementarity are apparent. Size markers were included in the far *left* and *right* lanes; their length is indicated in nt. An irrelevant shRNA (shNef) was included as a negative control. The regular shRNA 21-nt products are marked. (*) AgoshRNA ∼30-nt products (ranges from 25 to 35 nt). Ethidium bromide staining of small rRNAs and tRNAs are shown as loading controls *below* the blot. Similar results were obtained in an independent Northern blot experiment.

We next analyzed the RNA processing products by means of Northern blotting. Probing with a small oligonucleotide for the 3′ side of the molecule should detect the regular Dicer-processed guide strand of ∼21 bp (Fig. 4, top panel). Indeed, this fragment was abundantly present but only for shRNAs of 20 or more base pairs, which nicely correlates with the activity data. At the minimal size of 20 bp, it is evident that a top G-C base pair is beneficial for shRNA processing, consistent with the trend observed in the luciferase activity tests. We cannot formally exclude other processing paths that lead to products of ∼20 nt, e.g., a cellular nuclease that attacks the shRNA loop (Dallas et al. 2012), but deep sequencing did not point to such a possibility (Liu et al. 2013).

Ago-mediated processing should yield the typical AgoshRNA products of ∼30 nt (marked with an asterisk next to the Northern blot). The 5′ side probe will not only detect these AgoshRNA products but also the regular Dicer-cleaved passenger strand of ∼21 nt (Fig. 1B). As expected, these ∼21-nt molecules are the only products for the larger shRNAs of 21 bp and longer (Fig. 4, bottom panel). The hairpins of 20 or fewer base pairs show a hybrid pattern with both short Dicer and long Ago products. Most importantly, the Ago products gradually win over the Dicer products when the hairpins get shorter than 20 bp. The actual lengths of the predicted Dicer- and Ago-cleavage products are listed underneath Figure 2. The Northern blot shows the Ago products for the hairpins with a 15- to 20-bp stem, but they are lacking for larger templates. Most importantly, the Ago product is much enhanced for the G-U hairpins over the G-C variants. The 20-bp hairpin yields a complex but intriguing pattern: The G-U design favors the 35-nt AgoshRNA product, but the G-C variant favors the 20-nt Dicer product. An intense AgoshRNA product is visible for hairpins down to 17 bp, but the signal becomes less intense for even smaller hairpins, which is consistent with the results of the luciferase activity assays. Most of the cleavage products (∼21 and ∼30 nt) of the shortest duplexes (15 and 16 bp) disappear without a concomitant appearance of the precursor band (37 and 39 nt, respectively) as previously observed (Liu et al. 2013). These combined results demonstrate the importance of the duplex length and top G-U base pair for optimal AgoshRNA processing and activity. Although there might have been a small change for some of the constructs in transcription start site usage, we did not detect an effect on AgoshRNA expression levels.

### Further variation of the top base pairs

**FIGURE 5.**
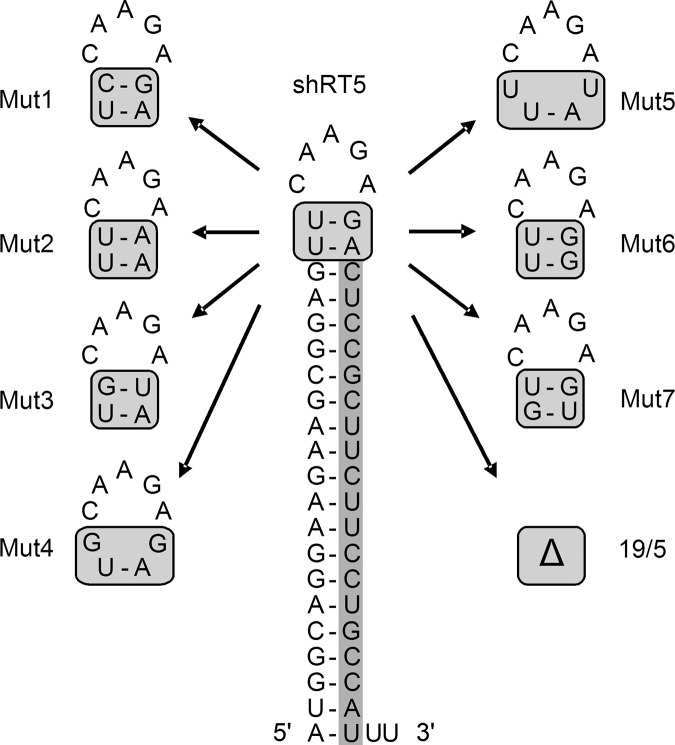
Design of shRT5 mutants with varying top base pairs. shRT5 with a 21-bp stem and 5-nt loop was used as the backbone. The encoded guide sequence targeting the Rev/Tat region of HIV-1 is boxed. The two top base pairs were modified or deleted (Δ).

The results obtained thus far indicate that the identity of the top base pair influences which processing route a shRNA molecule of intermediate stem size will use. The major routing determinant is the length of the base-paired stem, but fine-tuning is achieved by the top base pair, with the weak G-U base pair favoring the AgoshRNA route. We next made more variations in the top base pair to test whether this effect also holds true for the reverse U-G pair and for the penultimate base pair position. We specifically used the shRT5 template for this variation as this 21/5 hairpin has a hybrid shRNA/AgoshRNA character (Liu et al. 2013), which will allow us to detect small shifts in RNA routing. We realize that a shorter 19/5 shRNA construct will be a much better AgoshRNA design, but that choice would restrict the ability to identify variants with increased AgoshRNA activity. The 19/5 shRNA was included as a positive AgoshRNA control. The hairpin shRT5 has a top U-G (Fig. 5), which was stabilized by a point mutation in C-G (mutant 1) or U-A (mutant 2). The top base pair was also flipped into G-U (mutant 3), like the top base pair used in the first mutant set. We opened the top base pair by point mutation into the mismatches G.G (mutant 4) and U.U (mutant 5). We also created double U-G pairs by mutation of the second pair from U-A to U-G (mutant 6) or G-U (mutant 7). As explained above, these two top base pairs are formed by the universal loop sequences and are not part of the anti-sense/sense sequences designed to target a reporter gene.

**FIGURE 6.**
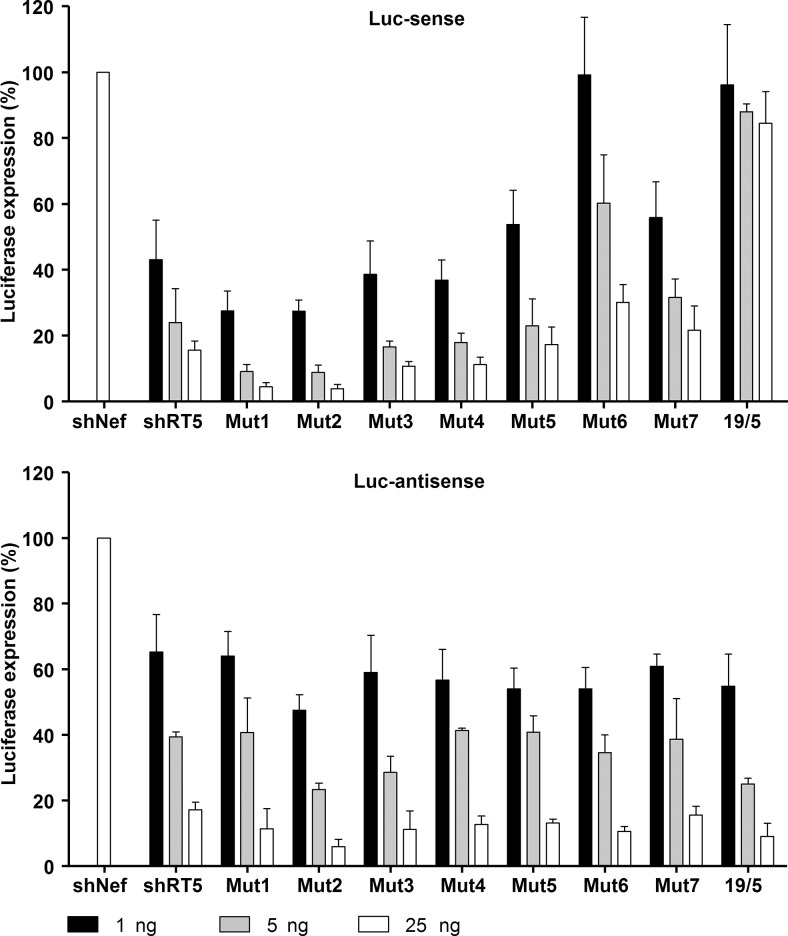
Knockdown activity of the shRT5 variants. The knockdown activity of the guide strand on Luc-sense (*upper* panel) and passenger strand on Luc-anti-sense (*lower* panel) of the shRT5 was determined by cotransfection of a luciferase reporter encoding the sense and anti-sense target sequence, respectively, in HEK 293T cells. We performed three independent transfections, each in duplicate, and standard deviations were calculated. See Figure 3 for details.

Luciferase assays were performed as described above to score the activity of the regular shRNA guide on the Luc-sense reporter (Fig. 6, upper panel). Activity scored in the presence of the unrelated shNef was set at 100%. The wild-type shRT5 construct shows good activity, with luciferase levels dropping to <20% with 25 ng of the inhibitor construct. Little effect was apparent for the modifications in mutants 3–5, but a modest increase in activity was seen for mutants 1 and 2 that stabilize the top base pair, whereas a loss of activity was measured for the G-U mutant 7 and, in particular, mutant 6. As expected, no activity was scored for the positive AgoshRNA control 19/5 on this reporter. This 19/5 AgoshRNA control did exhibit good activity on the Luc-anti-sense reporter (Fig. 6, lower panel). Compared to shRT5, the mutant constructs showed similar activity on the Luc-anti-sense reporter. Please realize that this reporter will detect the activity of both the AgoshRNA strand and the passenger strand of regularly Dicer-processed shRNAs (gray and white arrows in Fig. 1B, respectively).

**FIGURE 7.**
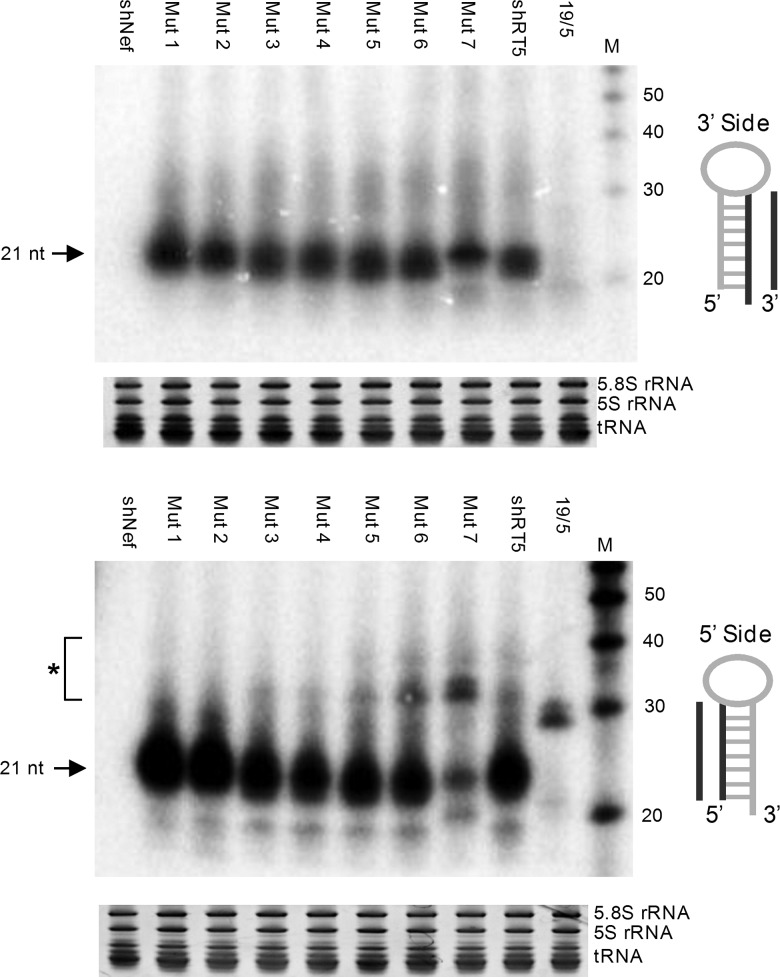
The type of top base pair influences shRNA processing. Five micrograms of the shRNA constructs was transfected in HEK 293T cells. Total RNA was analyzed by Northern blot with an LNA probe (see cartoon next to blot) for processing products derived from the 3′ strand (*upper* panel) and 5′ strand (*lower* panel). Small differences in probe-transcript complementarity are apparent. Size markers are indicated on the *right*. The regular shRNA ∼21-nt products are marked. (*) AgoshRNA ∼30-nt products. Ethidium bromide staining of small rRNAs and tRNAs are shown as loading controls *below* the blot. Similar results were obtained in two independent Northern blot experiments.

The Northern blot analysis revealed some striking differences among the mutant shRNAs. In particular, a significant loss of the regular 21 guide strand from the 3′ side (black arrow in Fig. 1) was apparent for mutant 7 (Fig. 7, top panel), reflecting the (moderate) loss of knockdown activity on the Luc-sense reporter. Mutant 6 demonstrates proper processing, but no impact on the knockdown activity was measured, which remains unexplained. The 5′ side probe detected both the AgoshRNA product of ∼30 nt and the passenger strand of ∼21 nt derived from regular shRNA processing (Fig. 7, lower panel). The most obvious difference is seen for mutant 6 and especially mutant 7, that produce a more prominent ∼30-nt signal via the Ago2 pathway (marked with an asterisk next to the Northern blot). The AgoshRNA control 19/5 generates exclusively the ∼30-nt product, which is, in fact, 4 nt shorter than the AgoshRNA products of the shRT5-derived mutants 6 and 7.

### The impact of Dicer knockdown

**FIGURE 8.**
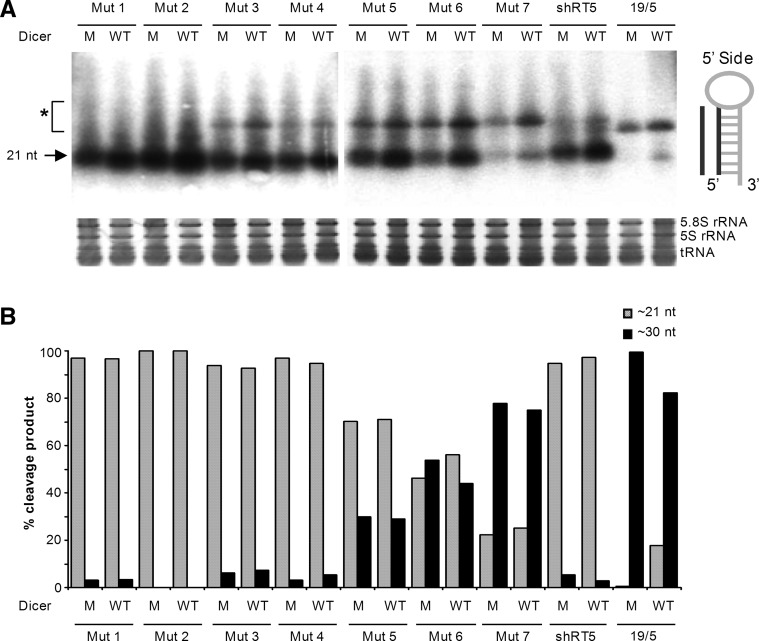
Processing of shRNA in DCR-deficient cells. (*A*) The indicated shRNA constructs (5 µg) were transfected in HCT116 wild-type (WT) or mutant (M) Dicer cells. Total RNA was isolated and analyzed by Northern blot analysis. HCT116 mutant Dicer cells encode an inactive Dicer with an interrupted helicase domain. The regular shRNA ∼21-nt products are marked. (*) AgoshRNA ∼30-nt products. Ethidium bromide staining of small rRNAs and tRNAs are shown as loading controls *below* the blot. (*B*) The Dicer and Ago2 cleavage products were quantified using ImageQuant. Total cleavage was set at 100%.

To directly probe the effect of Dicer on the processing of these shRNAs, we used the HCT116 cell line in which Dicer is knocked-down by disruption of the helicase domain (Cummins et al. 2006). We thus compared shRNA-processing patterns on a Northern blot in the presence of wild-type or mutant Dicer (Fig. 8, upper panel). The position of the regular Dicer products of ∼21 nt and the typical AgoshRNA product of ∼30 nt (*) are indicated. We quantitated these bands and plotted their relative levels (Fig. 8, lower panel). Although the AgoshRNA control 19/5 yields predominantly the ∼30-nt product, the minor ∼21-nt Dicer product was much reduced in the mutant Dicer background. A similar pattern was observed for mutant 7 and, to a lesser extent, for mutants 5 and 6, but not for mutants 1–4. It is possible that the intense and unexpected ∼21-nt product observed for mutants 1–4 in the mutant Dicer context reflects another RNA processing event, possibly 3′-end trimming of the ∼30-nt AgoshRNA product as described for other RNA molecules (Yoda et al. 2013). We also measured the knockdown activity on luciferase reporters. As expected, we maintained full AgoshRNA activity of 19/5 on the Luc-anti-sense reporters in these mutant Dicer cells (data not shown).

## DISCUSSION

We tested the hypothesis that a weak G-U base pair at the top of the shRNA stem is advantageous for using the noncanonical Ago2-processing route instead of regular Dicer-processing. Indeed, this effect was observed for several mutant shRNA sets in reporter assays and Northern blot analyses that discriminate between these two pathways. The Northern blotting was most informative by detection of the actual RNA cleavage products, being ∼21 nt and ∼30 nt for Dicer- and Ago2-mediated processing, respectively. Two possible explanations can be proposed for why the presence of a top G-U base pair favors the noncanonical AgoshRNA route. According to the first and most likely scenario, the weak top G-U base pair (Giese et al. 1998) may be opened transiently, thus presenting a stem that is actually 1 bp shorter, which impacts on the recognition by Dicer. In other words, the G-U effect may relate to the previously established and important rule of stem length for discriminating between the Dicer and AgoshRNA pathways (Liu et al. 2013). With a double set of weak G-U top base pairs as in mutants 6 and 7, the effective stem length may be 2 bp shorter by breathing of both base pairs. As a consequence, Dicer will ignore the 19-bp stem, whereas it would have been able to bind the 21-bp stem. As Dicer cleaves at a fixed distance from the base of the hairpin, this would obviously change the actual Dicer cleavage site, which could be probed by deep sequence analysis (Gu et al. 2012).

In addition to this thermodynamic scenario, one can propose a second, perhaps less likely, scenario, in which the weak G-U base pair plays a more active role. This G-U or U-G could either restrict recognition by Dicer or favor binding by Ago2. Some evidence in favor of this second scenario comes from the comparison of G-U variants with hairpins in which the top base pair is changed into a mismatch (mutants 4 and 5). These should benefit even more from the reduced stem length but were less active along the AgoshRNA route than the G-U variants. Future binding studies may be required to address these effects in further mechanistic detail.

We realize that the top base pair may be just one of the many sequence and structure elements probed by the Dicer and Ago2 endonucleases. Particular loop sequences may also influence the structural presentation of the top part of the hairpin. To probe this, we used the MC-Fold software, which assigns the most probable base-pairing interactions based on its frequency in known structures (Parisien and Major 2008). MC-Fold predicts cross-loop C-G pairing between the first and fourth base positions, which may affect molecular recognition by the Dicer enzyme. However, a similar 1–4 cross-loop pair is predicted for the less active mutants 1, 2, and 3. To complicate matters further, yet another cross-loop pair (2–7) is predicted for mutant 4 with the extended loop, and a double cross-loop pair (1–6, 2–5) is predicted for mutants 5 and 6, the latter at the expense of the terminal U-G pair. Although many details are lacking concerning the conformational aspects of a shRNA that trigger Dicer-restriction or Ago2-recognition, it seems safe to say that the stem length of the hairpin and—related to that—the presence of a top G-U base pair are the most important contributors.

The relative weakness of the top base pair may also influence a subsequent step in AgoshRNA processing: opening of the top half of the stem upon Ago2-mediated cleavage of the 3′ arm. For miR-451, oligouridylation at the 3′ end and subsequent 3′ trimming occurs to reach the mature miRNA length (Cheloufi et al. 2010; Cifuentes et al. 2010; Yang et al. 2010), and the poly(A)-specific endonuclease PARN was recently implicated (Yoda et al. 2013). Surprisingly, trimming was found not to be essential for subsequent target mRNA silencing in vitro and in vivo (Yoda et al. 2013). This correlates with our findings, suggesting that extended AgoshRNA cleavage products of ∼30 nt can execute efficient target mRNA silencing.

We think that this research is important not only for designing active and specific shRNA molecules but especially for the accurate design of efficient AgoshRNAs as a research tool or therapeutic molecules. For instance, the original shRNA design by Brummelkamp et al. has a short stem length of only 19 bp but appears to have been saved by the spontaneous formation of two additional base pairs using loop nucleotides, including the top G-U pair (Brummelkamp et al. 2002; Schopman et al. 2010). One variable needs further exploration. We sometimes mutated the first nucleotide of the shRNA transcript, which may affect the level of transcription from the H1 promoter. Based on the identity of +1 nucleotides in natural H1 transcripts, A and G were suggested as the best choices (Tuschl 2002). However, we scored more activity for the U/C transcripts (see, e.g., Fig. 3, Luc-sense, middle panel). Furthermore, we were mostly interested in the relative activity of regular shRNA vs. AgoshRNA molecules.

We previously listed the potential advantages of AgoshRNA reagents (Liu et al. 2013). In brief, it was suggested that alternatively processed AgoshRNAs may be more active in RNAi knockdown experiments than conventional shRNAs. More potent target knockdown was observed in the luciferase reporter assays with the 17- to 19-bp minimized AgoshRNA compared with the 21-bp regular shRNAs, but a direct comparison remains difficult as different active RNA strands are generated that are probed on different reporter constructs. Such optimized AgoshRNA therapeutics may allow one to reduce the RNA dosage, thus reducing the chance of adverse effects, e.g., due to saturation of the components of the RNAi pathway or due to off-target effects on unrelated mRNAs (Jackson et al. 2006). We also reasoned that AgoshRNAs can be improved further by increasing the base-pairing complementarity with the mRNA target by adaptation of the loop sequences. An obvious advantage of AgoshRNAs over regular shRNAs is the production of only a single active RNA strand, which will also reduce the chance of off-target effects. AgoshRNAs may have additional benefits as smaller hairpins than regular shRNAs. AgoshRNAs may exhibit a better safety profile concerning activation of the dsRNA-induced protein kinase R and interferon pathways (Pebernard and Iggo 2004). Because AgoshRNAs do not mature via Dicer, they will not compete with this miRNA biogenesis process, and AgoshRNAs seem attractive molecules to silence target genes in Dicer-deficient cells, e.g., monocytes that lack Dicer expression (Coley et al. 2010). Ago2-mediated processing may also yield more precise RNA molecules, as Dicer creates imprecise ends (Gu et al. 2012).

Some of the findings reported in this study are likely to be relevant also for our understanding of the processing of Dicer-independent miRNAs by Ago2. In fact, important parallels are apparent with the AgoshRNA field, especially the importance of the stem length and the presence of a weak top G-U base pair (Dueck et al. 2012). Inspection of the sequence and structure elements in other natural miRNAs that avoid Dicer-recognition may represent a versatile approach to direct future research.

## MATERIALS AND METHODS

### DNA constructs

The shRNA constructs were made by annealing complementary oligonucleotides (containing BamHI and HindIII sites) and inserting them into the BglII and HindIII sites of the pSUPER vector as previously described (Brummelkamp et al. 2002; Ter Brake et al. 2006; Schopman et al. 2010). The RNA secondary structure of the shRNA transcript was predicted by the Mfold web server (Zuker 2003). Firefly luciferase reporter constructs (pGL3; Promega) were made by insertion of a 50- to 70-nt HIV-1 sequence, with the 19-nt target region in the center, in the EcoRI and PstI sites of the pGL3 plasmid (Westerhout et al. 2005). The luciferase reporters with the sense and anti-sense target sequences were described previously (Liu et al. 2013). All DNA constructs were sequence-verified using the BigDye Terminator Cycle Sequencing kit (ABI). Hairpin RNA constructs were sequenced using a sample denaturation temperature of 98°C and upon addition of 1M betaine.

### Cell culture and DNA transfection

Human embryonic kidney (HEK) 293T and HCT116 cells were grown as a monolayer in Dulbecco's modified Eagle's medium (DMEM; Invitrogen) supplemented with 10% fetal calf serum (FCS) (Hybond), penicillin (100 units/mL), streptomycin (100 μg/mL), and minimal essential medium nonessential amino acids (DMEM/10% FCS) at 37°C and 5% CO_2_. For luciferase assays, HEK 293T and HCT116 cells were plated 1 d before transfection in 24-well plates at a density of 1.4 × 10^5^ cells per well in 0.5 mL DMEM/10% FCS without antibiotics. Cells were transfected with 100 ng of the firefly luciferase expression plasmid, 1 ng of *Renilla* luciferase expression plasmid (pRL), and 1, 5, or 25 ng of shRNA vector using Lipofectamine 2000 reagent (Invitrogen) according to the manufacturer's instructions. Cells were lysed 48 h post**-**transfection to measure firefly and *Renilla* luciferase activities using the Dual-Luciferase Reporter Assay System (Promega). The ratio between firefly and *Renilla* luciferase activity was used for normalization of experimental variations such as differences in transfection efficiencies. The empty vector pBluescript (pBS) and the irrelevant shRNA (shNef) served as negative controls. The ratio between the firefly and the *Renilla* luciferase activity in the presence of 25 ng of shNef was set at 100%. We performed three independent transfections, each in duplicate. The luciferase data were subsequently corrected for between-session variation as described previously (Ruijter et al. 2006). The resulting six values were used to calculate the standard deviation, shown as error bars.

### siRNA detection by Northern blotting

Northern blotting was performed as previously described (Liu et al. 2008, 2013). Briefly, HEK 293T or HCT116 cells were transfected with 5 μg of shRNA constructs using Lipofectamine 2000. Total cellular RNA was isolated after 48 h with the mirVana miRNA isolation kit (Ambion) according to the manufacturer's protocol. Isolated RNA was analyzed by denaturing 15%-polyacrylamide gel electrophoresis (precast Novex TBU gel, Invitrogen) using a [^32^P]-labeled Decade Marker (Ambion) for size estimation. To check for equal sample loading, the gel was stained with 2 µg/mL ethidium bromide for 20 min. De-staining was performed by rinsing the gel three times in water for 10 min. The ribosomal RNA (5S rRNA) and tRNA bands were visualized with UV light. The RNA was electro-transferred to a positively charged nylon membrane (Boehringer Mannheim, GmbH) and cross-linked to the membrane using UV (254 nm, 0.12 J). LNA oligonucleotide probes were 5′ end-labeled with the kinaseMax kit (Ambion) in the presence of 1 μL of [γ-^32^P]ATP (0.37 MBq/μL, PerkinElmer). We used the following oligonucleotides probes (LNA positions underlined) to detect the 5′ and 3′ strand of the siRNA, respectively: 5′-CTCCGCTTCTTCCTGCCAT-3′ and 5′-ATGGCAGGAAGAAGCGGAG-3′. The unincorporated nucleotides were removed on a Sephadex G-25 spin column (Amersham Biosciences). The blot was incubated in 10 mL ULTRAhyb hybridization buffer (Ambion) at 42°C for 30 min. After addition of the labeled LNA oligonucleotide, hybridization was performed at 42°C for 16 h. The blot was washed twice for 5 min at 42°C in 2× SSC/0.1% SDS and twice for 15 min at 42°C in 0.1× SSC/0.1% SDS and subsequently analyzed using a PhosphorImager (Amersham Biosciences) and the ImageQuant (v5.1) software package. Northern blot analysis was repeated at least twice.
